# Comprehensive evaluation of body composition in a wide age range of Iranian adults using bioelectrical impedance analysis: Tehran Lipid and Glucose Study

**DOI:** 10.1017/S1368980023002835

**Published:** 2024-01-02

**Authors:** Mahdieh Mohamadzadeh, Majid Valizadeh, Farhad Hosseinpanah, Amirabbas Momenan, Maryam Mahdavi, Maryam Barzin, Feridoun Azizi

**Affiliations:** 1 Obesity Research Center, Research Institute for Endocrine Sciences, Shahid Beheshti University of Medical Sciences, Tehran 19395-476, Iran; 2 Endocrine Research Center, Research Institute for Endocrine Sciences, Shahid Beheshti University of Medical Science, Tehran, Iran; 3 Prevention of Metabolic Disorders Research Center, Research Institute for Endocrine Sciences, Shahid Beheshti University of Medical Sciences, Tehran, Iran

**Keywords:** Body composition, Fat mass, Fat-free mass, Fat mass ratio (FMR)

## Abstract

**Objective:**

To evaluate sex- and age-stratified body composition (BC) parameters in subjects with wide age range of 20–79 years.

**Design:**

Cross-sectional.

**Setting:**

Participants of Tehran Lipid and Glucose Study (TLGS).

**Participants:**

Two thousand nine hundred seventy participants met our inclusion criteria. They were divided into five age groups, and BC parameters were analysed based on sex and age using a bioelectrical impedance analyser (BIA).

**Result:**

The mean age of the participants was 42·1 ± 12·5 years, and 54 % of them were males. The mean BMI was 26·7 ± 3·7 kg/m^2^. Obesity indices were significantly higher in females (*P* < 0·001); however, skeletal muscle mass (SMM) and fat-free mass (FFM) were significantly higher in males (*P* < 0·001). Both SMM and FFM decreased significantly after the age of 50 years. Obesity indices significantly increased from the age group of 20–29 to 30–39 years in males and the age groups of 30–39 to 40–49 years and 40–49 to 50–59 years in females. The fat mass ratio (fat mass/SMM) showed two peaks in both sexes (after the ages of 30 and 50 years in males and 40 and 50 years in females). A strong correlation was found between BMI and percentage of body fat (*r* = 0·823 in females *v*. *r* = 0·768 in males).

**Conclusion:**

This is the first community-based study in the MENA region identifying sex- and age-stratified BC values using BIA. Our findings can be used as a reference for comparison in appropriate settings.

Worldwide, the rising obesity rates pose a substantial public health concern due to its association with chronic illnesses, morbidity and mortality, imposing significant pressure on healthcare systems^(1,2)^. Currently, most assessments of overweight and obesity are based on the calculation of BMI due to its convenient evaluation method and established link with mortality^(3)^. However, BMI merely serves as an indicator of weight in relation to height (kg/m^2^), failing to offer insight into body composition (BC)^(4)^, and it overlooks key BC components like fat mass (FM) and fat-free mass (FFM)^(5)^. On the other hand, alterations in weight might indicate a blend of changes in FM and FFM, leading to diverse impacts on the risk of disease. In fact, research suggests that both FM and FFM might be more powerful indicators of disease risk compared to BMI^(6–9)^. For instance, various studies have indicated that fat mass percentage (%FM) is a superior predictor for risks associated with CVD^(6)^, serum lipid profiles^(8)^, breast cancer occurrence^(9)^ and coronary events^(7)^ in comparison with BMI. Other findings suggest that diminished skeletal muscle mass (SMM) or FFM, commonly known as sarcopenia, is linked to physical disability^(10)^, reduced physical performance^(11)^, insulin resistance, glucose intolerance^(12)^ and an elevated mortality risk^(13)^.

Bioelectrical impedance analyser (BIA) is a straightforward and precise approach for evaluating FM and FFM^(14)^, making it particularly valuable for extensive population-based research due to its cost-effectiveness, accessibility and user-friendliness^(5)^. Moreover, it is crucial to underscore that the process of measuring age-related BC changes alone will not remedy the issue. Instead, consistent monitoring of individuals over time can serve as a motivational catalyst for implementing interventions aimed at improving their situation or preventing declines. Additionally, the utilisation of population reference standards is invaluable for assessing individuals and gaining insights into how they compare. This comprehensive approach acknowledges that measurement alone is insufficient to address the underlying complexities associated with age-related BC changes^(5)^. Besides sex and age discrepancies, BC measurements differ among nations^(15–17)^ and ethnic groups^(18)^. So, globally, numerous epidemiological studies employing BIA have evaluated age, sex, and ethnic variations in FM and FFM, yielding conflicting outcomes. In the NHANES III study with 15 912 participants aged 12–80 years, males exhibited higher FFM than females, peaking at 60 years for males and 45–55 years for females before declining^(19)^. Conversely, a cross-sectional study of 5635 Swiss adults aged 15–98 years demonstrated that FFM reached its peak in males at 35–44 years and in females at 45–54 years before declining^(20)^. However, in both studies, females consistently exhibited higher FM across all ages, gradually rising throughout life for both sexes until old age, where it declined^(19,20)^. Despite its convenience and non-invasive nature, BIA’s precision may be influenced by hydration, body temperature and the choice of equations for analysis, as well as variations in validity across populations and device accuracy^(21)^.

Together, these findings emphasise probable population-specific distinctions in BC components. Therefore, in this study, we utilised a multi-frequency BIA device, the InBody 570, to assess BC parameters in Iranian subjects with wide age range of 20–79 years. The primary aim of this study was to establish standard measures for BC parameters in the Iranian adult population. By providing comprehensive and sex- and age-stratified data, our study sought to offer valuable reference values that can serve as a benchmark for health assessments and interventions in this specific demographic. These data are intended to aid healthcare professionals in diagnosing health risks related to BC, such as obesity and sarcopenia, and guiding personalised interventions for better health outcomes.

While BIA offers practical utility for large-scale epidemiological and clinical studies, it is imperative to recognise its limitations and potential discrepancies in accuracy across different population groups. We acknowledge that our findings are reliant on BIA measurements and urge future research to investigate the applicability and potential impact of different BIA modalities on BC assessment, particularly within specific populations such as Iranians.

## Materials and methods

### Study population

The Tehran Lipid and Glucose Study (TLGS) is a prospective survey on non-communicable diseases and related risk factors with follow-up intervals of 3 years. The design, rationale, data collection methods and sampling strategy of TLGS have been published earlier^(9)^. In this cross-sectional study conducted in phase VII (2019–2021) of TLGS, 5209 individuals (20–79 years old) who had BIA data were selected among 7986 participants via simple random sampling. The participants were selected based on strict inclusion and exclusion criteria, which considered various factors such as health status. We thoroughly assessed the demographic information to gain a comprehensive understanding of the participants’ overall health and well-being. Moreover, special attention was given to including only healthy individuals and excluding those with any underlying health conditions that could potentially confound the results. Individuals who had missing data (*n* 94), patients with diabetes (*n* 624), heart failure (*n* 53), renal failure (Glomerular Filtration Rate (GFR) < 60) (*n* 791), and history of cancer (*n* 29), pregnant (*n* 3) and lactating (*n* 34) women, those with BMI less than 18 (*n* 54) or over 35 (*n* 393), as well as people with a history of using diuretics (*n* 46) or glucocorticoids (*n* 112) were excluded from the study. Finally, 2970 participants were included in the final analysis. New cases of diabetes were not excluded from the study population for BC measurement.

Excluding individuals with new cases of diabetes could introduce selection bias and limit the generalisability of the findings to the broader population. The coexistence of diabetes and BC alterations, such as changes in FM and lean mass, is of clinical significance. Including individuals with new cases of diabetes allows for a comprehensive assessment of the relationship between diabetes and BC, which may be relevant for future studies and clinical practice.

The rationale behind excluding individuals with extreme BMI was made to enhance the accuracy and validity of our BC measurements and is a common practice in BC research.

An institutional ethics committee (i.e. the Research Institute for Endocrine Sciences affiliated with Shahid Beheshti University of Medical Sciences) approved the study (IR.SBMU.ENDOCRINE.REC.1400·062). All the tenets of the Declaration of Helsinki were followed, and all participants signed written informed consent.

### Measurements

Trained interviewers collected the data required, including demographic parameters, drug consumption and medical history information. All anthropometric measurements were performed following standard protocols.

Weight was measured using a digital scale (nearest 100 g), while the participant wore minimal clothes and no shoes. Height was determined using a tape measure, while the participant standing with no shoes and shoulders were in the standard straight position. Each participant’s height was measured three times, and the average of these replicate measurements was used for analysis to enhance measurement accuracy and reduce potential errors. BMI was defined as weight divided by height (kg/m^2^) and was used to categorise participants according to international cut-off points as normal (BMI < 25), overweight (25 ≤ BMI < 30) and obese (BMI ≥ 30). Waist circumference (WC) was recorded at the umbilicus level using a tape measure (to the nearest 0·1 cm) while imposing no pressure on the body surface.

BC was evaluated using a portable multi-frequency BIA device (model: InBody 570, InBody Co., Ltd). The InBody 570 is widely recognised for its validity and reproducibility in various populations, although its accuracy can be affected by factors such as hydration status, body temperature and population-specific traits. Our study’s BIA technique entailed the use of a portable multi-frequency BIA device with an eight-electrode configuration. Participants adhered to specific preparation instructions, including fasting for 2 h, avoiding caffeine for 2 h, refraining from exercise for 4–6 h, and wearing light clothing without shoes and socks. The BIA device passed a safe, low-level electrical current through the body, measuring impedance at multiple frequencies. Utilising predictive equations, it calculated various BC parameters, such as FM, percent body fat (PBF), FFM and SMM.

It should be noted that BIA is contraindicated in subjects carrying heart pacemakers, platinum, metal prosthesis or a Holter device in their bodies. Participants were instructed to remove any metals or jewellery they were wearing before undergoing the BIA measurement. This step was taken to minimise the potential interference and improve the reliability of the BIA results. Following wiping the palm and sole using an electrolyte tissue, participants stood on their soles touching the foot electrodes while their hands grabbed the hand-held paddle electrodes. Other data such as sex, height, weight and age were also recorded. BIA with eight electrodes assesses different segmental impedances (i.e. the trunk, right and left arms, and right and left legs) at 5, 50 500 kHz employing eight electrodes in a tetrapolar arrangement. Resistance against the alternating current was assessed while the individual was standing on the analyser’s platform. FM (in kg), PBF (% of fat in the body weight) and FFM (in kg) were obtained. The fat mass index (FMI, in kg/m^2^), fat-free mass index (FFMI, in kg/m^2^) and skeletal muscle mass index (SMMI, in kg/m^2^) were also calculated by dividing each value by the square of height. The fat mass ratio (FMR) was calculated by dividing the FM by SMM. The FMR is a valuable index that provides information about the balance between FM and muscle mass in the body. Its clinical relevance lies in its association with sarcopenic obesity, age-related changes in BC and its potential implications for cardiometabolic health.

The intraclass correlation coefficient (ICC) was used to analyse the reproducibility between the measures retrieved by the BIA device in each group^(22)^. The ICC is a statistical measure that evaluates the consistency or reproducibility of measurements, taking into account both technical reproducibility and biological day-to-day variation. Fifteen women and 16 men were selected as the sample based on relevant criteria. The same operator performed BC analyses twice (3 d apart) in each group. Men had a mean age of 24 ± 6·4 years, while women had a mean age of 35 ± 10·8 years. The ICC value and 95 % CI were determined using SPSS software version 20. The ICC and 95 % CI calculated for PBF and FFM were 0·996 (0·991, 0·998) and 0·998 (0·997, 0·999), respectively. The mean differences for the two measurements of FM and FFM were (0·04 ± 1·11) and (0·10 ± 1·04), respectively, indicating reliable values given their proximity to zero.

The algorithm employed in this study is based on predictive models integrating impedance, age, sex, height and weight to estimate BC parameters, with a foundation in regression analysis and insights from diverse populations, including those assessed by more accurate methods like Dual-Energy X-ray Absorptiometry (DXA)^(23,24)^.

BMR values were estimated using the BIA software, which employed prediction equations based on age, weight, height and sex. These values represent calculated estimates and should not be interpreted as direct measurements of BMR.

Following overnight fasting, blood sampling was performed, and the tests were conducted at the TLGS laboratory on the day on which blood sampling was done. Details of all the measurement methods are available elsewhere^(9)^.

### Definitions

According to Joint Interim Statement (JIS) criteria, obesity was defined based on the WHO’s BMI categories, and a WC cut-off point of ≥ 90 cm was designated as the abdominal obesity threshold in both sexes^(25)^. Excess PBF was expressed as PBF > 25 in men and > 35 in women^(26)^. Diabetes was defined as fasting plasma glucose ≥ 7·0 mmol/l or blood glucose > 11·1 mmol/l after Oral Glucose Tolerance Test with 75 grams of glucose (OGTT-75) gr or a history of taking antidiabetic drugs. The Chronic Kidney Disease Epidemiology Collaboration (CKD-EPI) formula was utilised to ascertain renal failure as GFR < 60 cc/min. Heart failure was defined as an ejection fraction of less than 45 % evidenced by echocardiography.

### Statistical analysis

Numerical continuous variables were presented as mean and standard deviation, and categorical variables were reported as absolute numbers and %. Based on a normal distribution, the mean values and the corresponding standard deviations of the continuous variables were considered. The means of normally distributed covariates were compared using the *t* test and ANOVA. Parameters were compared between age groups using the Bonferroni *post hoc* test. The *χ* ^2^ test was performed to analyse univariate statistical associations between categorical variables at the baseline. Percentile curves for sex-stratified BC parameters were generated using the least median of squares (LMS) model, and Pearson’s correlation was employed to assess the link between PBF, FFM, SMM and BMI. The Kappa value was used to check the agreement between BMI and PBF. Data analysis was performed in SPSS version 20, and a *P* value of < 0·05 was considered statistically significant.

## Results

Table 1 presents the participants’ baseline characteristics. This analysis included 2970 participants, aged 20–79 years (54 % men), who were categorised into five age groups (20–29 (*n* 472), 30–39 (*n* 928), 40–49 (*n* 754), 50–59 (*n* 535) and 60–79 (*n* 287)). Based on WHO criteria, the prevalence of overweight (BMI = 25–29·9) and obesity (BMI ≥ 30) were 44·2 % and 21·6 %, respectively. FM, PBF, FMR and visceral fat level (VFL) were significantly higher in women (*P* < 0·001), while SMM, FFM and BMR were significantly higher in men (*P* < 0·001).

**Table 1:** Basal characteristics of the study population

	Total (n 2970)			Males (n 1511)			Females (n 1459)			P
	Mean	sd	%	Mean	sd	%	Mean	sd	%	
Age, years	42·1	12·5		42·2	13·2		41·9	11·7		0·402
Height, cm	167·4	10·0		174·9	6·6*		159·6	6·2		<0·001
Weight, kg	75·1	13·7		82·5	12·6*		67·5	10·2		<0·001
BMI, kg/m^2^	26·7	3·7		26·9	3·6*		26·5	3·9		0·005
BMI categories										<0·001
NL			34·1			31·2			37·2	
Overweight			44·2			47·9*			40·2	
Obese			21·6			20·9			22·4	
WC, cm	92·4	10·0		94·9	9·9*		89·8	9·7		<0·001
WC										
≥90 cm			62·4			73·7*			51·2	<0·001
FM, kg	24·5	7·9		22·8	8·1		26·3	7·3		<0·001
FMI, kg/m^2^	8·9	3·2		7·5	2·7		10·4	3·0*		<0·001
PBF%	32·5	8·6		27·0	6·9		38·2	6·1		<0·001
PBF%										
>25 % (M) and 35 % (F)			65·1			60·5			69·8*	<0·001
SMM, kg	28·1	6·6		33·5	4·5*		22·5	2·8		<0·001
SMMI, kg/m^2^	9·9	1·4		10·9	1·0*		8·8	0·8		<0·001
FFM, kg	50·7	11·0		59·6	7·4*		41·5	4·7		<0·001
FFMI, kg/m^2^	17·9	2·2		19·4	1·7*		16·3	1·4		<0·001
FMR (FM/SMM)	0·92	0·36		0·68	0·24		1·17	0·30		<0·001
ECW ratio (ECW/TBW)	0·381	0·007		0·378	0·007		0·383	0·006		<0·001
Visceral fat level	11·3	4·5		9·9	4·2		12·7	4·3		<0·001
BMR, kcal/d	1465·2	236·7		1656·8	159·5*		1266·7	101·5		<0·001

WC, waist circumference; FM, fat mass; FMI, fat mass index; PBF, percent body fat; SMM, skeletal muscle mass; SMMI, skeletal muscle mass index; FFM, fat-free mass; FFMI, fat-free mass index; FMR, fat mass ratio; ECW ratio, extracellular water/total body water; NL, Normal Range or Reference Range.

Data are presented as mean ± sd or *n* (%).

We conducted statistical analyses to compare BC parameters between different age groups in addition to analysing differences based on sex. The participants were divided into five age groups: 20–29, 30–39, 40–49, 50–59 and 60+ years. We used these age groupings to examine differences in BC across various age groups, rather than assessing changes with age. As a whole, we found significant differences in obesity indices, including FM, PBF and FMR, between certain age groups in both males and females. Specifically, in men, we observed a significant increase in FM and PBF between the age groups of 30–39 years and 40–49 years (*P* < 0·05). Meanwhile, in women, significant increases in FM and PBF were noted between the age groups of 40–49 years and 50–59 years (*P* < 0·05).

Our analysis revealed a significant decrease in SMM and FFM after the age of 50 years in both males and females. In males, the mean SMM decreased from 34·4 kg (sd = 4·1) in the age group of 40–49 years to 32·3 kg (sd = 3·6) in the age group of 50–59 years, showing a decline of 2·1 kg. Similarly, the mean FFM decreased from 61·0 kg (sd = 6·8) to 57·7 kg (sd = 6·1), with a difference of 3·3 kg (Cohen’s d = –0·67). In females, the mean SMM decreased from 23·2 kg (sd = 2·6) in the age group of 40–49 years to 22·1 kg (sd = 2·4) in the age group of 50–59 years, indicating a decline of 1·1 kg. The mean FFM decreased from 42·6 kg (sd = 4·4) to 40·9 kg (sd = 4·1), with a difference of 1·7 kg. These findings indicate age-related alterations in muscle mass and FFM, with substantial reductions in SMM and FFM after the age of 50 years in both sexes.

Tables 2 and 3 compare anthropometric measures and BC parameters between different age groups in each sex. In all cases, there are statistical significant differences between the groups of age in Table 2 and 3 (*P* < 0·001 for all cases). Means of WC and BMI were significantly higher in the age group of 30–39 years compared to the age group of 20–29 years in both sexes. In addition, FM, PBF and VFL significantly increased by moving from 20–29 years of age to 30–39 years among men and by moving from 30–39 years of age to 40–49 and then from 40–49 to 50–59 years among women. In both sexes, SMM(I), FFM(I) and BMR remained unchanged from 20 until 50 years of age and decreased significantly afterwards. Two peaks were detected for FMR (FM/SMM) in both sexes (after the age of 30 and 50 years in men and after 40 and 50 years of age in women). The highest FMR values in both sexes were seen in the eldest. Among men, WC and BMI were stable after the age of 40 years and did not show significant changes with ageing; however, WC was significantly lower in the eldest participants. Nonetheless, WC steadily increased with age among women. In men, FM, PBF and VFL remained unchanged after the age of 40 years, while among women, these parameters gradually increased with age, especially after the ages of 40 and 50 years. Non-significant reductions were seen in FM and BMI in the eldest males and females. The extracellular water:total body water ratio was higher in men than in women and increased significantly from the age of 40 years onwards in both sexes.

**Table 2:** Anthropometric and body composition differences between age groups in males

	20–29 (*n* 256)			30–39 (*n* 480)			40–49 (*n* 330)			50–59 (*n* 275)			60+ (173)		
	Mean	sd	%	Mean	sd	%	Mean	sd	%	Mean	sd	%	Mean	sd	%
Height, cm	177·6	6·0		176·6	6·1		175·6	6·0		172·5	5·9		169·1	5·7	
Weight, kg	80·5	13·8		85·0	12·4		84·5	11·7		81·4	11·1		76·2	12·0	
WC, cm	89·9	10·6		95·6	10·1		95·9	9·0		96·6	8·3		95·2	10·0	
WC															
≥90 cm			51·6			75·3			80·6			82·5			71·1
BMI, kg/m^2^	25·5	3·9		27·2	3·5		27·4	3·3		27·3	3·3		26·6	3·7	
BMI categories															
NL			48·4			28·2			25·2			25·2			34·7
Overweight			37·5			50·2			50·3			52·9			44·5
Obese			14·1			21·5			24·5			21·9			20·8
FM, kg	19·5	9·1		23·5	8·1		23·4	7·4		23·6	7·5		23·2	7·9	
FMI, kg/m^2^	6·2	2·9		7·5	2·6		7·6	2·4		8·0	2·5		8·1	2·8	
PBF,%	23·3	7·9		27·1	6·6		27·2	6·0		28·5	6·2		29·6	6·8	
PBF% >25 %															
			42·2			60·7			60·9			69·0			72·8
SMM, kg	34·3	4·4		34·7	4·3		34·4	4·1		32·3	3·6		29·4	3·9	
SMMI, kg/m^2^	10·8	1·0		11·1	1·0		11·1	1·0		10·8	0·9		10·3	1·0	
FFM, kg	60·7	7·3		61·3	7·3		61·0	6·8		57·7	6·1		53·2	6·6	
FFMI, kg/m^2^	19·2	1·6		19·6	1·7		19·8	1·6		19·4	1·4		18·6	1·7	
FMR (FM/SMM)	0·56	0·25		0·68	0·23		0·68	0·21		0·73	0·23		0·79	0·25	
ECW ratio (ECW/TBW)	0·375	0·005		0·375	0·006		0·377	0·006		0·381	0·007		0·387	0·007	
Visceral fat level	8·1	4·5		10·1	4·1		10·1	3·8		10·4	4·1		10·7	4·3	
BMR, kcal/d	1680·9	157·7		1694·3	157·0		1688·7	147·7		1617·1	132·4		1519·6	142·6	

WC, waist circumference; FM, fat mass; FMI, fat mass index; PBF, percent body fat; SMM, skeletal muscle mass; SMMI, skeletal muscle mass index; FFM, fat-free mass; FFMI, fat-free mass index; FMR, fat mass ratio; ECW ratio, extracellular water/total body water; NL, Normal Range or Reference Range.

Data are presented as mean ± sd or *n* (%).

**Table 3:** Anthropometric and body composition differences between age groups in females

	20–29 (*n* 216)			30–39 (*n* 448)			40–49 (*n* 424)			50–59 (*n* 260)			60+ (*n* 114)		
	Mean	sd	%	Mean	sd	%	Mean	sd	%	Mean	sd	%	Mean	sd	%
Height, cm	162·4	6·3		161·0	5·6		159·9	5·8		156·7	5·4		154·3	6·0	
Weight, kg	63·7	10·8		66·2	10·5		69·4	9·3		70·2	9·2		66·5	9·5	
WC, cm	83·4	9·4		87·1	9·0		91·0	8·2		95·2	8·7		96·4	9·2	
WC															
≥90 cm			24·1			39·1			56·9			73·4			78·9
BMI, kg/m^2^	24·1	3·8		25·5	3·7		27·1	3·4		28·6	3·4		27·9	3·6	
BMI categories															21·1
NL			67·1			48·7			27·3			15·8			48·2
Overweight			22·7			35·9			49·3			44·8			30·7
Obese			10·2			15·4			23·5			39·4			
FM, kg	23·1	7·6		24·7	7·1		27·2	6·5		29·6	6·7		28·5	7·3	
FMI, kg/m^2^	8·8	2·9		9·5	2·7		10·7	2·6		12·1	2·7		12·0	3·1	
PBF,%	35·4	6·4		36·4	5·8		38·5	5·2		41·6	5·3		42·0	6·3	
PBF% >35 %															
			49·5			60·3			74·4			88·8			86·0
SMM, kg	22·2	3·0		22·8	2·9		23·2	2·6		22·1	2·4		20·5	2·4	
SMMI, kg/m^2^	8·4	0·8		8·8	0·9		9·1	0·8		9·0	0·7		8·6	0·7	
FFM, kg	40·9	5·0		41·9	4·8		42·6	4·4		40·9	4·1		38·4	4·1	
FFMI, kg/m^2^	15·5	1·3		16·2	1·4		16·6	1·3		16·6	1·2		16·1	1·1	
FMR (FM/SMM)	1·0	0·3		1·1	0·3		1·2	0·3		1·3	0·3		1·4	0·3	
ECW ratio (ECW/TBW)	0·382	0·005		0·381	0·005		0·383	0·006		0·386	0·006		0·391	0·007	
Visceral fat level	10·7	4·4		11·5	4·0		13·1	3·7		14·9	3·9		15·0	4·3	
BMR, kcal/d	1253·4	108·21		1275·2	104·0		1290·4	96·0		1253·8	88·7		1200·1	89·0	

WC, waist circumference; FM, fat mass; FMI, fat mass index; PBF, percent body fat; SMM, skeletal muscle mass; SMMI, skeletal muscle mass index; FFM, fat-free mass; FFMI, fat-free mass index; FMR, fat mass ratio; ECW/TBW, extracellular water/total body water; NL, Normal Range or Reference Range.

Data are presented as mean ± sd or *n* (%).

**Fig. 1:**
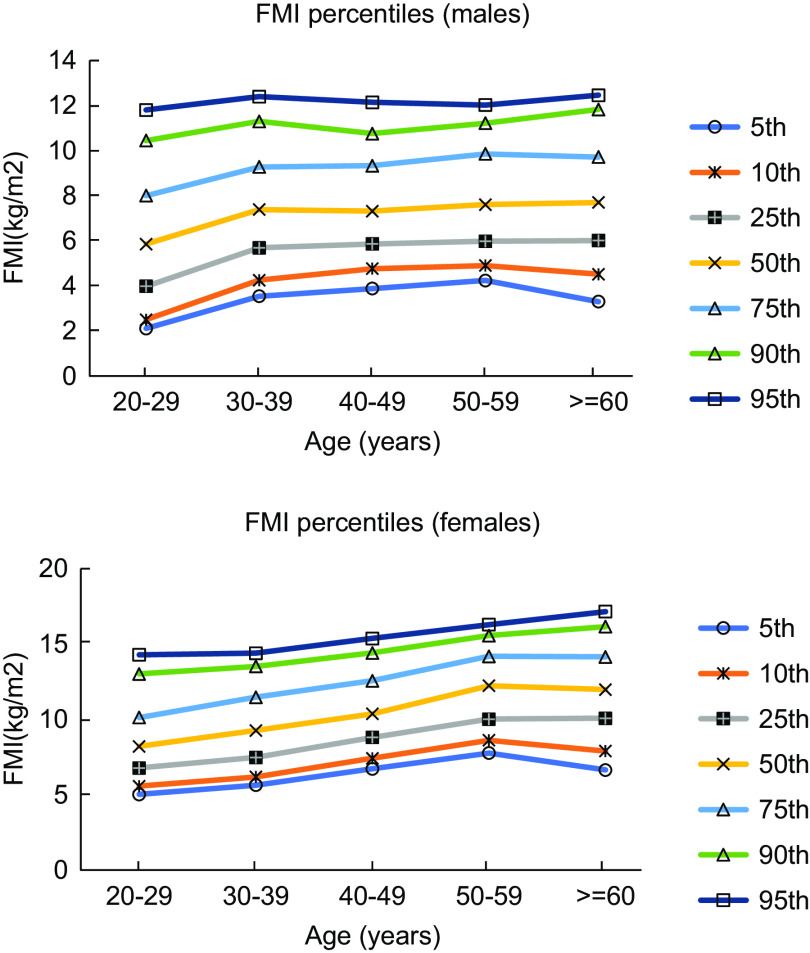
Percentile curves of FMI in different age groups in males and females. FMI, fat mass index

**Fig. 2:**
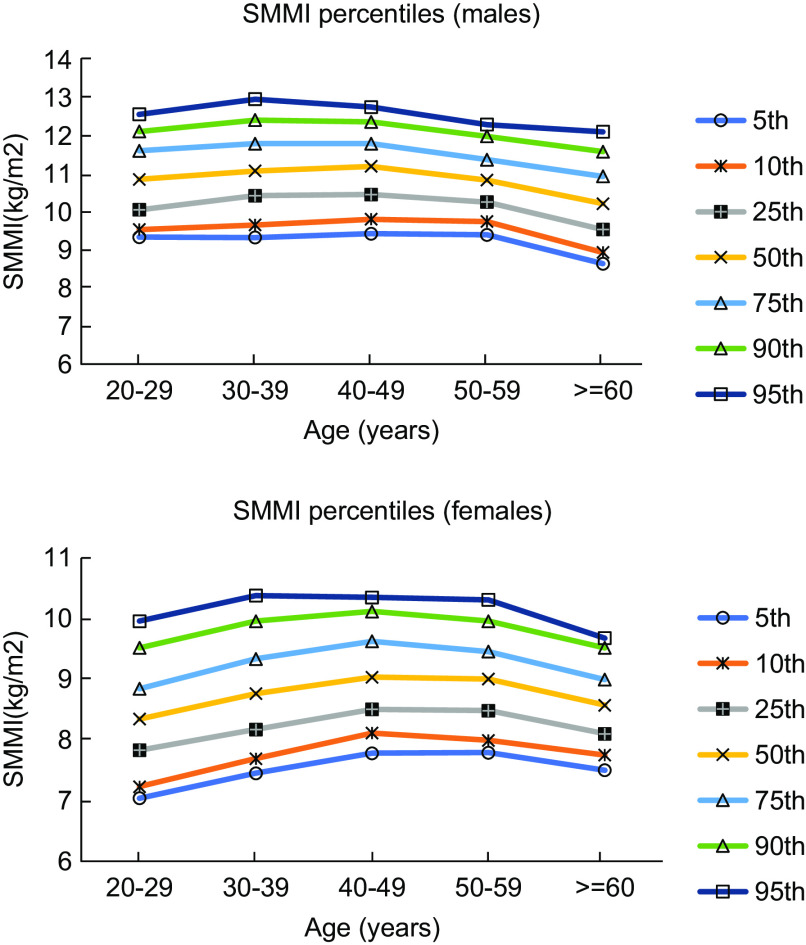
Percentile curves of SMMI in different age groups in males and females. SMMI, skeletal muscle mass index

Figures 1 and 2 show the percentile curves of FM and SMM in males and females aged 20–79 years. The reference ranges of FM (the 5–95th percentile) in the 20–29-year-old age group were 6·7–37·7 kg and 13·1–38·6 kg in males and females, respectively. Table 4 shows the reference ranges (5–95th percentile) of BC parameters in both sexes.

**Table 4:** Reference range (5–95 percentiles) of body composition parameters

Age groups (years)	20–29	30–39	40–49	50–59	60+
Men					
FM	6·7–37·7	11·2–37·8	11·9–37·3	12·3–36·0	10·3–36·9
PBF	10·4–36·8	16·4–38·0	17·2–37·5	18·4–37·8	17·7–40·4
SMM	27·5–42·0	28·1–42·2	27·9–41·3	26·8–38·9	23·9–36·7
FFM	49·4–73·9	50·3–74·2	50·1–72·8	48·3–69·2	44·2–65·6
FMR	0·2–1·1	0·3–1·1	0·4–1·1	0·4–1·1	0·4–1·2
Women					
FM	13·1–38·6	13·7–37·4	17·2–38·7	19·1–41·0	15·8–41·8
PBF	24·2–45·9	26·5–45·7	30·3–46·9	33·1–49·2	29·7–50·6
SMM	17·8–28·0	18·4–27·8	19·0–27·6	18·3–26·7	16·5–24·5
FFM	33·4–50·6	34·5–50·0	35·6–50·3	34·4–48·2	31·8–45·2
FMR	0·6–1·6	0·7–1·6	0·8–1·6	0·9–1·8	0·8–1·9

FM, fat mass; PBF, percent body fat; SMM, skeletal muscle mass; FFM, fat free mass; FMR, fat mass ratio.

**Fig. 3:**
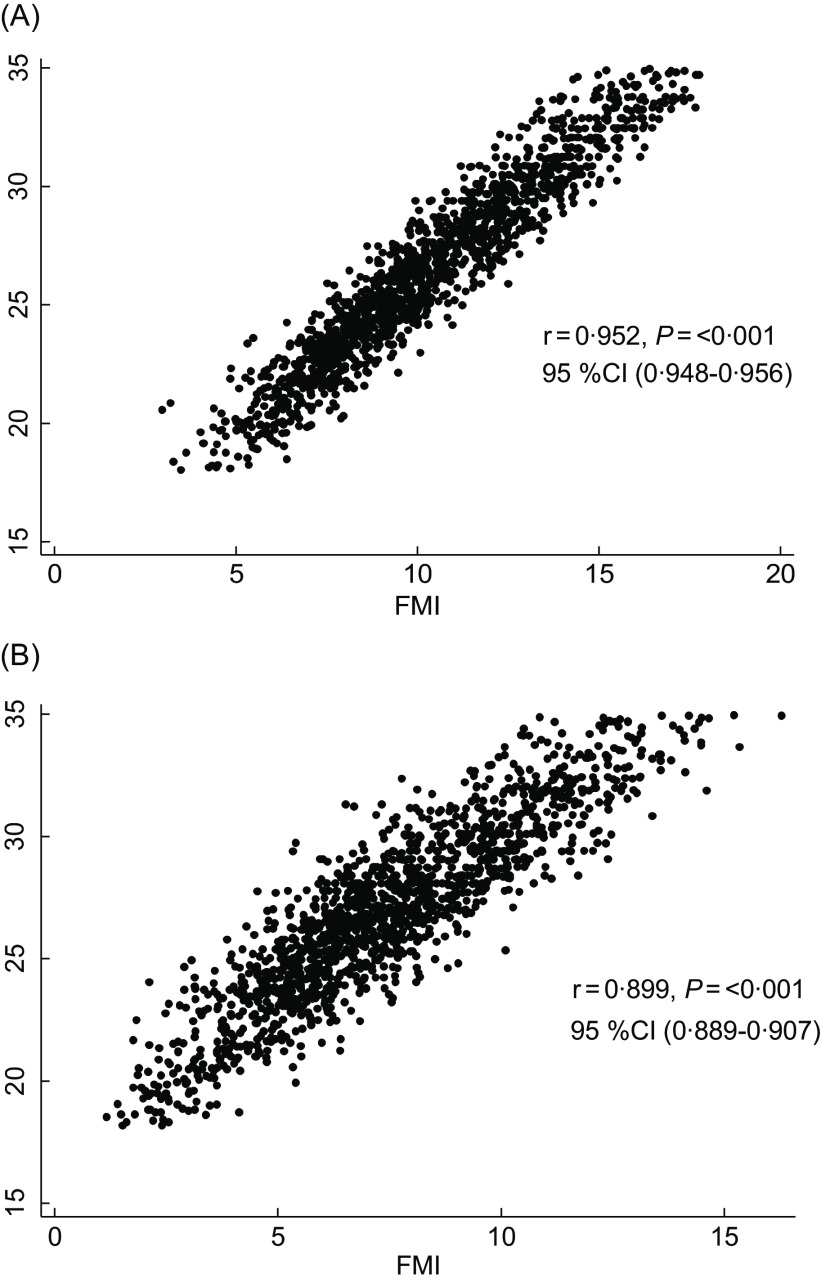
The correlation of BMI and FMI in female (A) and male (B). FMI, fat mass index

**Fig. 4:**
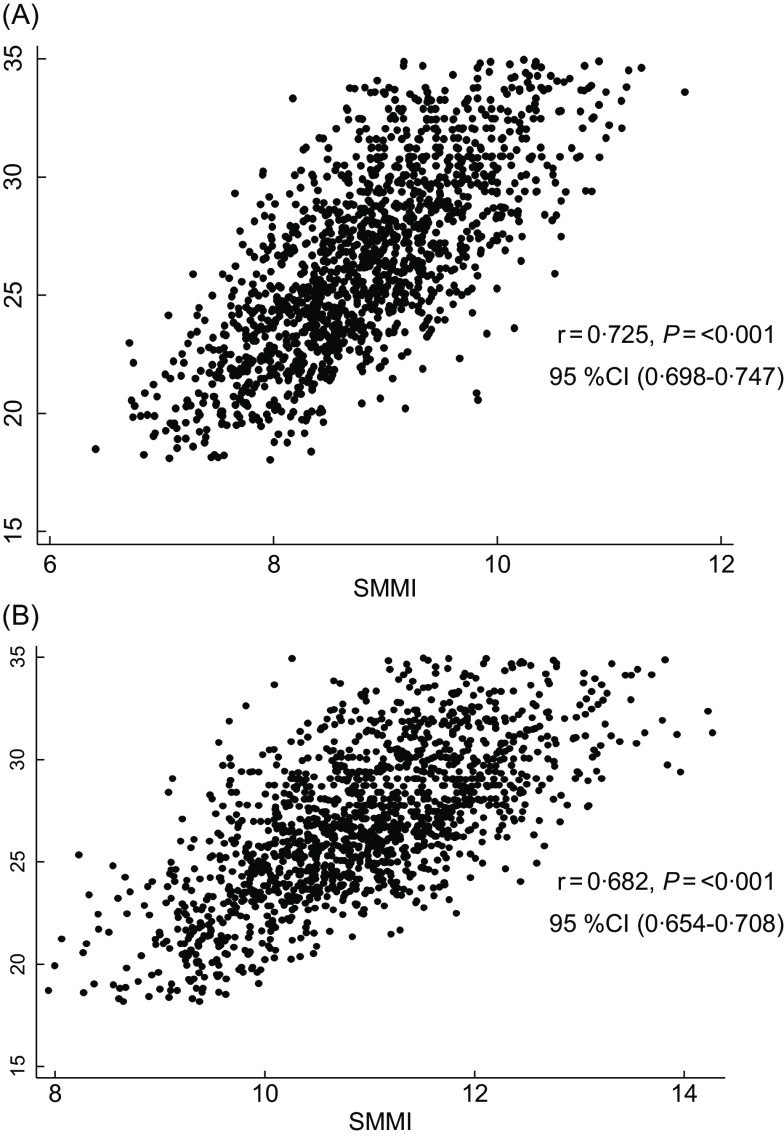
The correlation between BMI and SMMI in female (A) and male (B). SMMI, skeletal muscle mass index

In our study, we observed a strong correlation between BMI and PBF in females (*r* = 0·829, *P* < 0·001) and in males (*r* = 0·785, *P* < 0·001). Similarly, we noted a correlation between BMI and FFM as well as SMM in males (*r* = 0·486 for FFM, *P* < 0·001; *r* = 0·476 for SMM, *P* < 0·001) and in females (*r* = 0·418 for FFM, *P* < 0·001; *r* = 0·418 for SMM, *P* < 0·001). Figures 3 and 4 show the results of correlation analysis involving the BMI, FMI and SMMI parameters in both sexes.

## Discussion

This population-based cross-sectional study aimed to evaluate sex- and age-stratified BC parameters and percentile curves using BIA among healthy Iranian adults aged 20–79 years. Due to the limitations of BMI, especially its changes with age, numerous studies have tried to identify a variety of BC parameters and determine their reference values in their populations^(16,17,21,27)^. However, these values are specific to each race and cannot be applied to all regions. Due to the lack of a comprehensive nationwide study in Iran, we tried to address this issue and add to the current drawn picture in other countries. In our research, all participants in the study belong to the same ethnic group. Highlighting this fact will further strengthen the significance of our findings and their potential relevance to this specific ethnic population. The finding highlights that the study offers valuable reference values that could be valuable for comparing BC variations among Iranian urban populations. This resource enables healthcare professionals and researchers to evaluate individuals’ BC relative to age- and sex-specific norms. This aids in identifying health risks, such as low muscle mass or excessive fat, and guides personalised interventions. The provided data also have clinical applicability in diagnosing and managing health conditions related to BC, while serving as a foundation for further research into its impact on health outcomes within the Iranian population.

This study showed that women had higher FM, PBF, FMR and VFL than men, while SMM, FFM and BMR were higher in men than in women in all age groups. Both FM and PBF increased in males and females in the fourth and fifth decades of life, respectively, and a decrease was noticed in SMM and FFM after the age of 50 years in both sexes. Besides, FMR constantly increased with two noteworthy heights; the first coincided with a peak in FM (men: 30 years of age, women: 40 years of age), and the second coexisted with a decline in SMM and FFM (males and females: 50 years of age).

Even though in both males and females, FM and percentage grew in the fourth and fifth decades of life, a constant increase was only seen in women. However, FMR (FM/SMM) persistently increased with ageing in both sexes. Similar to our findings, in the NHANES III study, FMR (i.e. the FM/FFM ratio) was reported to be higher in women, and its highest value was recorded in the age group of 60–69·9 years in both sexes. This index was suggested to be a more comprehensive criterion for assessing BC alterations compared to any other indicator^(28)^. According to a 2021 study by Zhang *et al.* on China’s rural population, FMR increased steadily with age in both sexes, while FMI remained stable up to the age of 50 years in women and 70 years in men^(29)^. The main limitation of BMI, as mentioned earlier, is that it cannot differentiate between changes in FM or FFM. Our observation highlighted the importance of FMR in identifying FM elevation in conjunction with muscle mass loss when BMI remains unchanged.

Similar to our research, various studies have noted a strong correlation between BMI and PBF^(30–33)^. In the present study, the correlation between BMI, SMM and FFM was moderate. This finding shows that the diagnostic performance of BMI is not optimal for identifying the loss of FFM. We found that 25·7 % of individuals with normal BMI (18·6 % of men and 31·8 % of women) had a BF% within the obesity range. Given the proposed role of normal-weight obesity in boosting the risk of CVD^(34)^, it is recommended to analyse BC parameters along with BMI for conducting a more accurate cardiometabolic risk assessment.

In comparison with other populations, Zhang *et al.*, in a study on rural people (2021, China)^(29)^, reported lower FM and FMR in both sexes compared to our study. Similar to our study, they noticed an increase in FM with ageing (i.e. over the age of 50 years in women and the age of 70 years in men), which was accompanied by a decrease in SMM in older people (beyond the age of 65 years). Slight variabilities between these two studies can be attributed to different lifestyles adopted by rural and urban populations. Similarly, Xue He *et al.* in another study in China^(17)^ underlined that villagers had a more stable FM upon ageing. This difference highlights the importance of racial, genetic and lifestyle parameters as determinants of BC. This prompts a thoughtful consideration of the possibility that the rural Chinese population may exhibit more favourable or healthier BC values than those observed in the current study. The mean values of BMI, FM and FFM obtained in our study were comparable with those reported by Franssen *et al.* ^(16)^. Decreased BMI and FM in both sexes and lower WC in men, which were observed in the eldest people, are consistent with the fact that these parameters gradually fall in people older than 70 years. As well, the small sample size or the higher mortality rate in people with elevated FM in this age category can be other reasons for this observation. Consistently, in the NHANES study, FM was reported to decline in people over the age of 70 years^(28)^.

Age-related changes in BC can have significant implications, particularly in relation to sarcopenia and undernutrition diagnoses. Sarcopenia and undernutrition are two important conditions that often occur in older adults and can adversely affect their health and quality of life. Understanding the age-related changes in BC can provide valuable insights into these conditions and help in their early diagnosis and management. Assessing age-related changes in SMM is crucial for the early diagnosis of sarcopenia. In our study, we observed a significant decrease in SMM after the age of 50 years in both males and females. This finding aligns with previous research that has shown a decline in muscle mass with advancing age. By providing age-specific data on SMM in our population, our study can serve as a valuable reference for diagnosing sarcopenia. Clinicians can use the age-specific data to establish cut-off points for low muscle mass in older adults. Identifying individuals with low muscle mass early on can facilitate timely interventions, such as resistance training and adequate protein intake, to prevent or mitigate the progression of sarcopenia.

Age-related changes in BC play a pivotal role in nutritional health. Loss of muscle mass associated with ageing can impair physical function and mobility, leading to reduced food intake and potential undernutrition. Increased FM may contribute to metabolic disturbances, impacting appetite regulation and nutrient utilisation. Our study identified noteworthy obesity indices increases after specific age thresholds, emphasising the importance of monitoring BC for accurate nutritional assessments. The obtained data are crucial for diagnosing undernutrition, establishing age-specific reference values and enabling timely interventions. Recognising the significance of age-related BC changes is vital for diagnosing and managing conditions like sarcopenia, contributing to improved health in our community.

The main strength of our study is that it is the first community-based research providing age- and sex-stratified BC parameters in Iran and the MENA region. This study provides valuable insights into sex- and age-stratified BC parameters in 20–79-year-old Iranian adults using BIA. The use of multi-frequency BIA in our study offers distinct advantages over other methods, enhancing the precision and comprehensiveness of BC assessments. By capturing information at various frequencies, this approach provides a more accurate analysis of intra- and extracellular water, ensuring reliability and reproducibility, especially in scenarios with varying hydration levels. The targeted analysis of specific tissues or compartments further contributes to the method’s superior resolution, distinguishing it as a more precise and comprehensive option compared to other BIA approaches. However, the relatively smaller sample size in the older age group may affect the precision of estimates. Exclusion of individuals with extreme BMI may limit generalisability, and the accuracy of BIA equations may vary for different ethnicities, including Iranians. While BIA is practical, its limitations in estimating BC in certain populations should be considered. The absence of a reference method like DXA^(35)^ warrants caution. While BIA equations can provide reasonably accurate estimations of BC in the population for which they were developed, they may not fully account for all variations in BC due to ethnicity, race or other factors. The validity of BIA measurements can be influenced by factors such as hydration status, body shape and variations in body tissues’ electrical properties, which can differ among individuals of different ethnic backgrounds. Despite these limitations, the study provides valuable data for comparing BC variabilities among Iranian adults, contributing to clinical assessments and evaluations of health consequences. Emphasising the limitations of BIA as a method for assessing BC in Iranian adults is important for interpreting results accurately.

### Conclusion

This was the first population-based study reporting sex- and age-stratified BC values in a group of healthy subjects with wide age range 20–79 years in our region. These values can be used as a reference for further comparisons with other populations inside and outside of Iran to draw more accurate conclusions on their clinical applicability and health-related outcomes. Obesity indices and muscle mass were higher in men than in women. Obesity parameters were observed to significantly rise in the age group of 30–39 years in men. In contrast, women showed a significant increase in these determinants one decade later (i.e. the age over 40 years). Muscle mass deteriorated significantly in both sexes from 50–59 years of age onward. Further studies are required to evaluate the relationship between these parameters and their predictive value for cardiometabolic health.

## References

[1] Finucane MM , Stevens GA , Cowan MJ et al. (2011) National, regional, and global trends in body-mass index since 1980: systematic analysis of health examination surveys and epidemiological studies with 960 country-years and 9·1 million participants. Lancet 377, 557–567. 10.1016/S0140-6736(10)62037-5 PMC4472365 21295846

[2] Wang YC , McPherson K , Marsh T et al. (2011) Health and economic burden of the projected obesity trends in the USA and the UK. Lancet 378, 815–825. 21872750 10.1016/S0140-6736(11)60814-3

[3] World Health Organization (2000) *Obesity: Preventing and Managing the Global Epidemic. Report of a WHO Consultation. World Health Organization Technical Report Series 894*. Geneva: WHO. pp. i–xii, 1–253. 11234459

[4] National Health and Medical Research Council (NHMRC) (2003) Clinical Practice Guidelines for the Management of Overweight and Obesity in Adults. Canberra: NHMRC.

[5] Kyle UG , Bosaeus I , De Lorenzo AD et al. (2004) Bioelectrical impedance analysis--part I: review of principles and methods. Clin Nutr 23, 1226–1243. 15380917 10.1016/j.clnu.2004.06.004

[6] Marques-Vidal P , Bochud M , Mooser V et al. (2009) Obesity markers and estimated 10-year fatal cardiovascular risk in Switzerland. Nutr Metab Cardiovasc Dis 19, 462–468. 19185476 10.1016/j.numecd.2008.10.001

[7] Calling S , Hedblad B , Engstrom G et al. (2006) Effects of body fatness and physical activity on cardiovascular risk: risk prediction using the bioelectrical impedance method. Scand J Public Health 34, 568–575. 17132589 10.1080/14034940600595621

[8] Nagaya T , Yoshida H , Takahashi H et al. (1999) Body mass index (weight/height2) or percentage body fat by bioelectrical impedance analysis: which variable better reflects serum lipid profile? Int J Obes Relat Metab Disord 23, 771–774. 10454113 10.1038/sj.ijo.0800961

[9] Lahmann PH , Lissner L , Gullberg B et al. (2003) A prospective study of adiposity and postmenopausal breast cancer risk: the Malmo Diet and Cancer Study. Int J Cancer 103, 246–252. 12455040 10.1002/ijc.10799

[10] Baumgartner RN , Koehler KM , Gallagher D et al. (1998) Epidemiology of sarcopenia among the elderly in New Mexico. Am J Epidemiol 147, 755–763. 9554417 10.1093/oxfordjournals.aje.a009520

[11] Newman AB , Kupelian V , Visser M et al. (2003) Sarcopenia: alternative definitions and associations with lower extremity function. J Am Geriatr Soc 51, 1602–1609. 14687390 10.1046/j.1532-5415.2003.51534.x

[12] Kalyani RR , Metter EJ , Ramachandran R et al. (2012) Glucose and insulin measurements from the oral glucose tolerance test and relationship to muscle mass. J Gerontol A Biol Sci Med Sci 67, 74–81. 21350243 10.1093/gerona/glr022 PMC3260481

[13] Wannamethee SG , Shaper AG , Lennon L et al. (2007) Decreased muscle mass and increased central adiposity are independently related to mortality in older men. Am J Clin Nutr 86, 1339–1346. 17991644 10.1093/ajcn/86.5.1339

[14] Bosy-Westphal A , Later W , Hitze B et al. (2008) Accuracy of bioelectrical impedance consumer devices for measurement of body composition in comparison to whole body magnetic resonance imaging and dual X-ray absorptiometry. Obes Facts 1, 319–324. 20054195 10.1159/000176061 PMC6452160

[15] Liu P , Ma F , Lou H et al. (2013) The utility of fat mass index *v.* body mass index and percentage of body fat in the screening of metabolic syndrome. BMC Public Health 13, 1–8. 23819808 10.1186/1471-2458-13-629 PMC3703297

[16] Franssen FM , Rutten EP , Groenen MT et al. (2014) New reference values for body composition by bioelectrical impedance analysis in the general population: results from the UK Biobank. J Am Med Dir Assoc 15, 448-e1. 10.1016/j.jamda.2014.03.012 24755478

[17] He X , Li Z , Tang X et al. (2018) Age- and sex-related differences in body composition in healthy subjects aged 18–82 years. Medicine 97, e11152. 29924020 10.1097/MD.0000000000011152 PMC6023800

[18] Liu B , Du Y , Wu Y et al. (2021) Trends in obesity and adiposity measures by race or ethnicity among adults in the United States 2011–2018: population based study. BMJ 372, n365. 33727242 10.1136/bmj.n365 PMC7961695

[19] Chumlea WC , Guo SS , Kuczmarski RJ et al. (2002) Body composition estimates from NHANES III bioelectrical impedance data. Int J Obes Relat Metab Disord 26, 1596–1609. 12461676 10.1038/sj.ijo.0802167

[20] Kyle UG , Genton L , Hans D et al. (2001) Age-related differences in fat-free mass, skeletal muscle, body cell mass and fat mass between 18 and 94 years. Eur J Clin Nutr 55, 663–672. 11477465 10.1038/sj.ejcn.1601198

[21] Jin M , Du H , Zhang Y et al. (2019) Characteristics and reference values of fat mass index and fat free mass index by bioelectrical impedance analysis in an adult population. Clin Nutr 38, 2325–2332. 30389251 10.1016/j.clnu.2018.10.010

[22] Koo TK & Li MY (2016) A guideline of selecting and reporting intraclass correlation coefficients for reliability research. J Chiropr Med 15, 155–163. 27330520 10.1016/j.jcm.2016.02.012 PMC4913118

[23] Malavolti M , Mussi C , Poli M et al. (2003) Cross-calibration of eight-polar bioelectrical impedance analysis *v.* dual-energy X-ray absorptiometry for the assessment of total and appendicular body composition in healthy subjects aged 21–82 years. Ann Hum Biol 30, 380–391. 12881138 10.1080/0301446031000095211

[24] Demura S , Sato S & Kitabayashi T (2004) Percentage of total body fat as estimated by three automatic bioelectrical impedance analyzers. J Physiol Anthropol Appl Hum Sci 23, 93–99. 10.2114/jpa.23.93 15187381

[25] Azizi F , Khalili D , Aghajani H et al. (2010) Appropriate waist circumference cut-off points among Iranian adults: the first report of the Iranian National Committee of Obesity. Arch Iran Med 13, 243–244. 20433230

[26] De Lorenzo A , Bianchi A , Maroni P et al. (2013) Adiposity rather than BMI determines metabolic risk. Int J Cardiol 166, 111–117. 22088224 10.1016/j.ijcard.2011.10.006

[27] Lee MM , Jebb SA , Oke J et al. (2020) Reference values for skeletal muscle mass and fat mass measured by bioelectrical impedance in 390 565 UK adults. J Cachexia Sarcopenia Muscle 11, 487–496. 10.1002/jcsm.12523 PMC7113534 31943835

[28] Xiao J , Purcell S , Prado C et al. (2018) Fat mass to fat-free mass ratio reference values from NHANES III using bioelectrical impedance analysis. Clin Nutr 37, 2284–2287. 29056283 10.1016/j.clnu.2017.09.021

[29] Zhang J-X , Li J , Chen C et al. (2021) Reference values of skeletal muscle mass, fat mass and fat-to-muscle ratio for rural middle age and older adults in western China. Arch Gerontol Geriatr 95, 104389. 33713879 10.1016/j.archger.2021.104389

[30] Vanavanan S , Srisawasdi P , Rochanawutanon M et al. (2018) Performance of body mass index and percentage of body fat in predicting cardiometabolic risk factors in Thai adults. Diabetes Metab Syndr Obes 11, 241. 29910627 10.2147/DMSO.S167294 PMC5987789

[31] Ranasinghe C , Gamage P , Katulanda P et al. (2013) Relationship between body mass index (BMI) and body fat percentage, estimated by bioelectrical impedance, in a group of Sri Lankan adults: a cross-sectional study. BMC Public Health 13, 1–8. 24004464 10.1186/1471-2458-13-797 PMC3766672

[32] Amani R (2007) Comparison between bioelectrical impedance analysis and body mass index methods in determination of obesity prevalence in Ahvazi women. Eur J Clin Nutr 61, 478–482. 17063145 10.1038/sj.ejcn.1602545

[33] Romero-Corral A , Somers VK , Sierra-Johnson J et al. (2008) Accuracy of body mass index in diagnosing obesity in the adult general population. Int J Obes 32, 959–966. 10.1038/ijo.2008.11 PMC2877506 18283284

[34] Khonsari NM , Khashayar P , Shahrestanaki E et al. (2022) Normal weight obesity and cardiometabolic risk factors: a systematic review and meta-analysis. Front Endocrinol 13, 857930. 10.3389/fendo.2022.857930 PMC8987277 35399938

[35] Yang SW , Kim TH & Choi HM (2018) The reproducibility and validity verification for body composition measuring devices using bioelectrical impedance analysis in Korean adults. J Exerc Rehabil 14, 621. 30276183 10.12965/jer.1836284.142 PMC6165971

